# Supplementary material for: Soft-Tissue, Rare Earth Element, and Molecular Analyses of Dreadnoughtus schrani, an Exceptionally Complete Titanosaur from Argentina
Source: Biology (Basel). 2022 Aug 2;11(8):1158. doi: 10.3390/biology11081158 (PMC9404821; doi:10.3390/biology11081158)
Supplement: Supplementary file 1 [file biology-11-01158-s001.zip › Schroeter et al., 2022 - Supplementary Materials.pdf]

Soft-tissue, rare earth element, and molecular analyses of *Dreadnoughtus schrani*, an exceptionally complete titanosaur from Argentina

Elena R. Schroeter[1], Paul V. Ullmann[2], Kyle Macauley[2], Richard D.F Ash[3], Wenxia Zheng[1], Mary H. Schweitzer[1], Kenneth J. Lacovara[2]

[1]Department of Biological Sciences, North Carolina State University, Raleigh, NC 27695, USA; easchroe@ncsu.edu, wzheng2@ncsu.edu, mhschwei@ncsu.edu

[2]Department of Geology, Rowan University, Glassboro, NJ 08028, USA; ullmann@rowan.edu, macauleyk4@students.rowan.edu, lacovara@rowan.edu

[3]Department of Geology, University of Maryland, College Park, MD 20742, USA; rdash@umd.edu

1. Supplemental Methods

1.1. Laser ablation-inductively coupled plasma mass spectrometry (LA-ICPMS)

We employed the same mass spectrometry methods as Ullmann et al (2020). Briefly, D. schrani fragment was embedded under vacuum in Silmar resin. A Hillquist SF-8 trim saw was used to cut a thick section (~3 mm), which was rinsed with distilled water and allowed to thoroughly dry. Specimen was subjected to LA-ICPMS using a New Wave UP-213 (213 nm wavelength) Nd:YAG laser coupled to a Finnigan Element2 ICPMS at the University of Maryland. All elemental concentrations are reported in parts per million (ppm) except for iron, which is reported in weight percent (wt. %). The laser was operated at 2–3 J/cm2 and a pulse rate of 7 Hz, and NIST 610 glass was used as an external standard. After background collection for 20 s, transect data were acquired using a laser diameter of 30 µm moving at 50 µm/s. Elemental concentrations were calculated based on normalization to 55.8% CaO in bone apatite. Reproducibility (taken as the percent relative standard deviation of all REE in NIST 610 glass) averaged 1.5% and was below 3% for every element except iron (6.2%).

1.2. Demineralization and Evaluation of Morphological Structures in Fossil Bone

Fragments of *D. schrani* humerus were incubated in 0.5 M disodium ethylenediaminetetraacetic acid (EDTA) (pH 8.0) for two weeks. Once bone tissue became pliable, portions of the tissue and supernatant were transferred onto a glass slides using sterilized pipettes. Although effort was made to obtain a humeral fragment that had not been exposed to either polyvinyl acetate (Vinac) or cyanoacrylate (Paleo Bond), acetone was applied directly to demineralization products immediately prior to imaging to completely rule out glue pseudomorphs as a source for the observed structures. Direct application of acetone to recovered structures (instead of incubating the bone fragment in acetone prior to demineralization) was used to ensure maximum exposure of these structures to the reagent. Demineralization products were imaged in transmitted light and cross-polarized light using a Zeiss Axiocam MRC5 camera mounted to a Zeiss Axioskop 2 Plus biological microscope and a Zeiss Axioskop petrographic polarizing microscope, respectively. Low magnification images were acquired on a Zeiss Stemi 2000-C dissecting microscope.

Table S1. Fossil demineralization protocol. Fossil bone was demineralized to (1) release potential soft-tissue structures (e.g., cells, vessels) from bone matrix for examination, and (2) to release demineralized bone matrix for evaluation by immunofluorescence (IF).

Sample Type	Location	Step	Reagent	Volume	Incubation Time @ Temp	Notes
Fossil Bone Only	*Pieces of fossil bone were added to the wells of a 6-well plate (Corning Inc.).					
Fossil Bone Only	6-well plate	Demineralization	0.5 M EDTA (pH 8.0; 0.22µm filtered)	~10 mL/well	14 days @ RT	EDTA changed ~daily

1.3. Defleshing and degreasing of extant bone

Table S2. Extant bone defleshing and degreasing protocols. Extant bone (chicken, alligator) were defleshed and degreased in preparation for molecular analyses. These steps were not necessary for fossil specimens.

Sample Type	Step	Location	Reagent	Volume	Incubation Time @ Temp	Reps	Notes
Extant Bone Only	Degreasing	Beaker w/ stir bar	10% Shout OR 10 % Zout (in 18.2 MΩ UltraPure H_2O)	500 mL	2-3 days @ RT	-	with stirring
Extant Bone Only	*Bone is removed from degreasing solution and sectioned into pieces.						
Extant Bone Only	Rinsing	Beaker w/ stir bar	18.2 MΩ UltraPure H_2O	500 mL	30 min @ RT	3x	with stirring

1.4. Immunofluorescence

1.4.1. Fixation & Demineralization of Extant Bone

Table S3. Extant bone fixation and demineralization protocols. Extant bone (chicken, alligator) was fixed prior to demineralization for immunofluorescence. This step was omitted for fossil samples because the cross-links induced by formalin fixation, while preserving tissue integrity, also cause loss of immunoreactivity that is only partially recoverable (Werner et al., 2002). Because signal loss was not a concern for extant tissues that have abundant protein content, fixation was performed only on these specimens.

Sample Type	Location	Step	Reagent	Volume	Incubation Time @ Temp	Reps	Notes
Extant Bone Only	*A sectioned piece of degreased, extant bone was fixed prior to demineralization.						
Extant Bone Only	1.5 mL tube	Fixation	10% Neutralized Formalin (in 1X PBS)	1 mL	overnight @ 4˚C	1x	-
Extant Bone Only	1.5 mL tube	Rinsing	1X PBS[a]	1 mL	10 min @ RT	3x	-
Extant Bone Only	*Fixed bone is transferred to 50 mL tube for demineralization						
Extant Bone Only	50 mL tube	Demineralization	0.5 M EDTA (pH 8.0; 0.22µm filtered)	~50 mL	~10 days @ RT	-	EDTA changed ~daily
Extant Bone Only	50 mL tube	Rinsing	1X PBS	~50 mL	10 min @ RT	3x	-
Extant Bone Only	*Soft, demineralized bone was sectioned into very small pieces with a razor.						
	a: Phosphate buffered saline solution						

1.4.2. Tissue Embedding

Demineralized *D. schrani* tissue was transferred to 1.5 mL tubes, washed with 18.2 MΩ water, and dehydrated in 70% ethanol. Samples were then incubated in a solution of 70% ethanol and LR White™ resin, followed by incubation in undiluted resin. Tissue was then transferred into 0.95 mL gelatin capsules, which were filled with LR White™ resin and polymerized at 60˚C for two days. Extant samples of alligator and chicken tibia were embedded in LR White™ in a separate, identical procedure, with the exception that they were fixed in 10% formalin prior to embedding (see 1.4.1. above). This step was omitted for fossil samples because the cross-links induced by formalin fixation, while preserving tissue integrity, also cause loss of immunoreactivity that is only partially recoverable (Werner et al., 2002). Because signal loss was not a concern for extant tissues that have abundant protein content, fixation was performed only on these specimens.

Table S4. Tissue embedding protocol. Fossil and extant bones were embedded in resin for subsequent thin sectioning and analyses with immunofluorescence. Fossil and extant bones were embedded and analyzed in separate labs.

Sample Type	Location	Step	Reagent	Volume	Incubation Time @ Temp	Reps	Notes
All	*Small pieces of demineralized tissue is transferred to 1.5 mL centrifugation tube						
All	1.5 mL tube	Washing	18.2 MΩ UltraPure H_2O	1 mL	10 min @ RT	6x	
All	1.5 mL tube	Dehydration	70% ethanol (in 18.2 MΩ H2O)	1 mL	30 min @ RT	2x	in fume hood
All	1.5 mL tube	Resin Infusion	LR White resin: 70% ethanol (2:1)	1 mL	1 hour @ RT	1x	in fume hood
All	1.5 mL tube	Resin Infusion	100% LR White resin	1 mL	1 hour @ RT	2x	in fume hood
All	*After final incubation in 100% resin, tissue is transferred to a 0.95 mL gelatin capsules (size "00"; 8.81 mm diameter, EMS)						
All	0.95 mL capsule	Polymerization	100% LR White resin	~1 mL	2 days @ 60˚C	1x	in oven

1.4.3 Immunofluorescence Assay

Sections of embedded tissue 220 nm thick were taken using an ultramicrotome (Leica EM UC6) equipped with separate ultra diamond knives (DiATOME®) for ancient and extant tissues. Sections were dried to a Teflon-printed slide, incubated in 0.5 M EDTA (for antigen retrieval), then washed in phosphate buffered saline (PBS) designed for immunohistochemistry (Zheng & Schweitzer, 2012) (hereafter, I'PBS). Autofluorescence was quenched with sodium borohydride ($NaBH_4$), then sections were washed with I'PBS. Tissues were "blocked" by incubation in 4% normal goat serum (4% NGS) to inhibit non-specific binding. After blocking, sections received one of three treatments: (1) incubation in "primary" antibody, polyclonal rabbit anti-chicken collagen I (1:40); (2) incubation in "primary" antibody that had been inhibited with chicken collagen (10 mg/mL) (this step performed in extant lab for all samples); or (3) incubation in 4% NGS with no primary antibody (i.e. "secondary only"). After incubation,

sections were washed with I'PBS+Tween20, then pure I'PBS, followed by incubation in "secondary" antibody, biotinylated goat anti-rabbit IgG H+L (1:500, Vector). After washing, sections were incubated in a fluorescent label, fluorescein isothiocyanate (FITC; Fluorescein Avidin D; Vector; 1:1000), then washed a final time prior to mounting with anti-fade mounting medium and a coverslip. Sections were imaged using a Zeiss Axiocam MRC5 camera mounted to a Zeiss Axioskop 2 Plus microscope. All sections were imaged at an exposure of 150 ms. To ensure all pixels of all images were identically adjusted, fluorescent (FITC) images for all tissues were compiled into a single image, then simultaneously converted to grey scale and adjusted for brightness, contrast, and gamma using PhotoShop CS5.

Table S5. Immunofluorescence protocol. Fossil and extant bone sections were subjected to immunofluorescence assays and simultaneous specificity controls (inhibition and secondary only controls, which test for nonspecific paratopes in a polyclonal antibody and nonspecific binding of the secondary antibody, respectively).

Sample Type	Step	Reagent	Volume	Incubation Time @ Temp	Reps	Notes
All	*Tissue sections (220 nm thick) were cut from embedded resin bullets with an ultramicrotome (Leica EM UC6) equipped with an Ultra Diamond Knif**e** (DiATOME®)					
All	*Multiple tissue sections (7-10 per well) were transferred to the wells of a 6-well Teflon printed glass slide.					
All	Drying	(this step adheres sections to slide)	-	overnight @ 45˚C	1x	covered, on a hot plate
All	Antigen retrieval	0.5 M EDTA (pH 8.0; 0.22µm filtered)	75 µL /well	20 min @ RT	4x	in a sealed container
All	Washing	1X I'PBS[a]	~100 mL	5 min @ RT	3x	in a Coplin jar; with rocking
All	Quench Autofluorescence	$NaBH_4$ (1 mg/mL in 18.2 MΩ UltraPure H2O	75 µL /well	10 min @ RT	3x	in a sealed container
All	Washing	1X I'PBS[a]	~100 mL	5 min @ RT	3x	in a Coplin jar; with rocking
All	Blocking	4% Normal Goat Serum in 1X I'PBS[a] (4% NGS)	75 µL /well	2-4 hr @ RT	1x	in a humid chamber
Selections of each sample type received each of the 3 treatments	Primary Antibody<br><br>**US Biological, C7510-13B*	**(1)** polyclonal rabbit anti-chicken collagen I* (1:40 in 4% NGS) **[OR]**<br>**(2)** polyclonal rabbit anti-chicken collagen I* (1:40 in 4% NGS) inhbited with purified chicken collagen (10 mg/mL) **[OR]**<br>**(3)** 4% NGS only	75 µL /well	overnight @ 4˚C	1x	in a humid chamber
All	Washing	1X I'PBS[a] + 5% Tween20	~100 mL	10 min @ RT	3x	in a Coplin jar; with rocking
All	Washing	1X I'PBS[a]	~100 mL	10 min @ RT	3x	in a Coplin jar; with rocking
All	Secondary Antibody	biotinylated goat anti-rabbit IgG H+L (1:500 in 1X I'PBS; Vector BA-1000)	75 µL /well	2-4 hr @ RT	1x	in a humid chamber
All	Washing	1X I'PBS[a] + 5% Tween20	~100 mL	10 min @ RT	3x	in a Coplin jar; with rocking

All	Washing	1X I'PBS[a]	~100 mL	10 min @ RT	3x	in a Coplin jar; with rocking
All	Labeling	fluorescein isothiocyanate (FITC)[b] (1:1000 in 1x I'PBS[a])	75 µL /well	1 hr @ RT	1x	in the dark; in a humid chamber
All	Washing	1X I'PBS[a] + 5 % Tween20	~100 mL	10 min @ RT	3x	in a Coplin jar; with rocking
All	Washing	1X I'PBS[a]	~100 mL	10 min @ RT	3x	in a Coplin jar; with rocking
All	Mounting	VectaShield H-1000 Anti-Fade Mounting Medium for Fluorescence	10 µL /well	-	-	cover slip applied after
All	*Sections were imaged using a Zeiss Axiocam MRC5 camera mounted to a Zeiss Axioskop 2 Plus microscope					
	a: Phosphate buffered saline formulated for immunohistochemistry, (Zheng & Schweitzer, 2012)<br>b: Fluorescein Avidin D; Vector A-2001					

1.4.4 Extended Digestion & Immunofluorescence Assay

Digestion assays were performed as described in 1.4.3 above with the following additions: before antigen retrieval with EDTA, select sections were digested with collagenase A (Roche; 1mg/mL in Delbucco's PBS) over three distinct time ranges: 24 hours, overnight, or 1 hour. Additional sections that were not subjected to digestion with collagenase were simultaneously taken through all other steps of the digestion procedure, to establish a baseline of non-enzymatic degradation. After final digestion, slides were washed in I'PBS, and protocol proceeded as detailed above, starting with antigen retrieval in EDTA.

Table S6. Digestion protocol Digestion Sections of fossil bone sections were subjected to varying lengths of digestion with collagenase A, to test whether observed antibody binding patterns were affected by the targeted destruction of collagen I. Digestions included: **(1)** 24 hours with 5 changes and then 1 hour with 3 changes (24 hr), **(2)** overnight with 1 change and then for 1 hour with 3 changes (O/N), **(3)** 1 hour with 3 changes only (1 hr), or **(4)** no collagenase (control).

Sample Type	Step	Reagent	Volume	Reagent Application Length	Reagent Changes	Slide Incubation Time @ Temp	Reps	Notes
All	*Tissue sections (220 nm thick) were cut from embedded resin bullets with an ultramicrotome (Leica EM UC6) equipped with an Ultra Diamond Knife (DiATOME®)							
All	*Multiple tissue sections (7-10 per well) were transferred to the wells of a 6-well Teflon printed glass slide.							
All	Drying	(this step adheres sections to slide)	-	-	-	overnight @ 45˚C	1x	covered, on a hot plate
Selection of Each	Extended Digestion	collagenase A (Roche) (1mg/mL in 1X D'PBS[a])	75 µL /well	**[24 hr]:** 24 hr<br>**[O/N]:** overnight<br>**[1 hr]: -**<br>**[control]: -**	**[24 hr]:** 5x<br>**[O/N]:** 1x<br>**[1 hr]: -**<br>**[control]: -**	24 hr @ 37˚C	1x	in a humid chamber

Selection of Each	Digestion	collagenase A (Roche) (1mg/mL in 1X D'PBS[a]	75 µL /well	**[24 hr]:** 1 hr **[O/N]:** 1 hr **[1 hr]:** 1hr **[control]:** -	**[24 hr]:** 3x **[O/N]:** 3x **[1 hr]:** 3x **[control]:** -	1hr @ 37˚C	1x	in a humid chamber
All	Washing	1X I'PBS[b]	~100 mL		N/A	5 min @ RT	2x	in a Coplin jar; with rocking
All	*After washing, protocol proceeded as detailed in "Immunofluorescence" table (Table S6) above.							
All	*Note: The different treatments above represent different wells on the same slide. Reagent application varied between wells in terms of length of time and number of applications, but the entire slide was incubated as shown in the "slide incubation" column.							
	a: Delbucco's phosphate buffered saline with calcium and magnesium<br>b: Phosphate buffered saline formulated for immunohistochemistry, (Zheng & Schweitzer, 2012)							

1.5 Enzyme Linked Immunosorbent Assay (ELISA)

1.5.1 Chemical Extraction of Proteins

Bone (2g) and sediment (2g) fragments were ground with sterilized mortar and pestles (extant bone was frozen for 20 sec in liquid nitrogen prior to grinding). Bone and sediment powders were added to 10 mL Zeba spin columns (Thermo Scientific); an additional spin column was left empty to serve as a buffer 'blank' control. Spin columns were placed in 50 mL tubes (Fisherbrand) and 10 mL of 0.6 M hydrochloric acid (HCl) was added to each column before incubation at RT overnight. Columns were transferred to collection tubes and centrifuged. The resulting supernatant, hereafter 'HCl extraction,' was removed stored at 4˚C. Samples then received 10 mL of 4M guanidine hydrochloride (GuHCl) followed by incubation at 65˚C overnight. After centrifugation, the resulting supernatant (hereafter 'GuHCl extraction') was stored at 4˚C. Following storage, extracted HCl and GuHCl supernatants were centrifuged to pellet any residual bone debris, then transferred to clean 50 mL tubes. Proteins were precipitated from HCl extracts by incubation with 100% trichloroacetic acid (TCA) for 1 hour at 4˚C. GuHCL extracts were precipitated by incubation with 100% ethanol overnight at -20˚C. After precipitation, HCl and GuHCl extractions were centrifuged, and supernatant was decanted and discarded. Extraction pellets were gently washed with 100% acetone (HCl extracts) or 90% ethanol (GuHCl extracts). Centrifugation and wash steps were repeated for both pellets, then supernatants were decanted by tube inversion, and pellets were allowed to dry overnight inverted in a laminar flow hood. Once dry, tubes were stored at -80˚C until analysis.

Table S7. Protein extraction protocol. Fossil, sediment, “buffer” blank, and extant bone samples were subjected to chemical extraction to solubilize bone proteins for subsequent molecular assays.

Sample Type	Location	Step	Reagent	Volume	Incubation Time @ Temp	Centri-fugation	Reps	Notes
All	*2g cortical bone fragments or sediment were coarsely ground (grain sizes ~1–2 mm diameter) in a mortar and pestle that sterilized by washing in nitric acid and baking at 520˚C overnight.							
Extant Only	*Extant bone fragments were frozen for 20 sec in liquid nitrogen prior to grinding.							
All	*Bone/sediment powders were added to empty 10 mL Zeba spin columns (Thermo Scientific). An additional spin column was left empty to serve as a buffer ‘blank’ control.							
All	*Columns were added to 50 mL polypropylene centrifuge tubes (Fisherbrand).							
All	Spin column	Demineral-ization	0.6 M hydrochloric acid (HCl)	10 mL	overnight @ RT	-	1x	with agitation @ 45˚ angle
All	Spin column	HCl Supernatant Collection	-	-	1 min	1000 rcf	1x	spin column moved to clean collection tube
All	Spin column	Protein Solubilization	4M guanidine hydrochloride (GuHCl) in 0.05 M Tris (~pH 7.4)	10 mL	overnight @ 65˚C	-	1x	with rocking @ 45˚ angle
All	Spin column	GuHCl Supernatant Collection	-	-	1 min	1000 rcf	1x	spin column moved to clean collection tube
All	*Collected HCl extracts ([HCl]) and GuHCl extracts ([GuHCl]) were stored in 50 mL centrifugation tubes @ 4˚C until protein precipitation.							
All	50 mL tube	Pelleting Supernatant Debris	-	-	10 min	8000 rcf	1x	supernatant transferred to new tubes, debris discarded
All	50 mL tube	Protein Precipitation	**[HCl]:** 100% Trichloroacetic acid (1.428 g TCA/ 1 mL H2O) **[GuHCl]:** 100% ethanol	2.5 mL<br>25 mL	1 hr @ 4˚C<br>overnight @ -20˚C	-	1x	
All	50 mL tube	Pelleting Precipitated Protein	**[HCl]:**<br>**[GuHCl]:**	-	20 min<br>10 min	8500 rcf	1x	supernatant decanted & discarded
All	50 mL tube	Pellet Washing	**[HCl]:** 100% acetone **[GuHCl]:** 90% ethanol	5 mL	20 min<br>10 min	8500 rcf	2x	each wash = add reagent, centrifuge, decant
All	*After final wash, tubes were inverted in a laminar flow hood and allowed to dry overnight. Dried pellets were stored @ -80˚C until analyses.							

1.5.2. ELISA

GuHCl extraction pellets from sediment and fossil bone were resuspended in PBS. Because no pellet was visible in negative control (“blank”) samples, PBS was pipette directly into extraction tube and recollected to obtain any trace extraction products. Chicken and alligator GuHCl extracts were diluted in PBS to the concentration of 5-10 µg/mL.

For each ELISA assay, a selection of wells on a 96-well U-bottom microtiter plate (Thermo Scientific) received 100 µL of resuspended samples, 100 µL of PBS (group blank), or no sample (plate blank) and were allowed to incubate for 2.5–4 hrs. Next, nonspecific binding of the wells was inhibited with 5% bovine serum albumin (5% BSA) incubated for 4 hrs at RT. A selection of wells from each of the sample types was then incubated overnight at 4˚C with one of the following treatments: (1) incubation in “primary” antibody, polyclonal chicken specific anti-collagen I (1:400), or (2) 5% BSA only. Plate was washed in ELISA wash buffer, then all wells were incubated for 2.5–4 hrs at RT with 100 µL of “secondary” antibody (1:2000; alkaline phosphatase conjugated goat anti-rabbit IgG (H+L)). The plate was again washed with ELISA wash buffer, and 100 µL of colorimetric substrate was added to each well. Absorbance was measured at 405 nm with a Molecular Devices Spectra Max Plus microplate reader (ancient samples) or a Molecular Devices THERMOmax microplate reader (extant samples). Data were acquired in Softmax Pro 4.8.

Table S8. ELISA protocol. Extraction products from fossil, sediment, "buffer" blank, and extant bone samples were subjected to ELISA. Specific details about how extraction products were resuspended for analyses, and in what proportions, is listed below.

Fossil & Sediment Samples Only	*To minimize loss and sample handling, entire extraction pellets derived from fossil bone and sediment were resuspended in PBS to created a stock solution that was aliquoted and diluted for assays. Dilutions for the these sample are expressed in terms of the weight of the original bone powder represented by the portion of the resuspened material used. ELISA analyses plated extraction stock representing the extraction products of 62.5 mg to 150 mg of fossil/sediment powder per well (6.25 - 15 mg/mL). (Note: all wells for fossils and sediment received the same portion of material in each trial. Listed range is between separate replication trials).					
Buffer (Blanks) Only	*Because no extraction pellet resulted from buffer/blank specimens, the same amount of 1X PBS used to resuspend the fossil and sediment samples was pippette into the buffer extraction tubes, vortexted, centrifuged, and recollected to ensure that any minute portion of extraction products (including any contaminants) were collected and tested as part of the negative controls.					
Extant Bone Only	*Because extant bone produced an abundance of extraction production, precisely measured dilutions could be created (in contrast to the fossil/sediment samples). Extant bone samples were resuspended in 1X PBS to concentrations that ranged between 5 µg/mL - 10 µg/mL over all trials (100 µL aliquots contained 500 ng - 1 µg extraction products). Concentations were consistent between wells in each trial, but varied across replication trials.					
**Sample Type**	**Step**	**Reagent**	**Volume**	**Incubation Time @ Temp**	**Reps**	**Notes**
All	*Solutions were plated on a 96-well Immulon 2HB U-bottom microtiter plate (Thermo Scientific). (Assays of fossil and extant bone were performed in separate labs on different days. Fossil and negative controls were plated on same plate and tested simultaneously.					
All	Antigen Plating	**[Fossil/Sediment]**: extract products from 6.25 mg - 15 mg bone powder per 1 mL 1X PBS<br>**[Buffer]**: diluted identically to fossil/sediment samples with 1X PBS<br>**[Extant bone]:** 5-10 µg/mL	100 µL /well	2.5-4 hr @ RT	1x	plate covered with titer top
All	Blocking	5% bovine serum albumin diluted in 1X PBS with 0.05% Thimersol and 0.005% Tween20 (5% BSA)	200 µL /well	4 hr @ RT	1x	plate covered with titer top
All	Primary Antibody	**(1)** polyclonal rabbit anti-chicken collagen I* (1:400 in 5% BSA) **[OR]**<br>**(2)** 5% BSA only<br>**US Biological, C7510-13B*	100 µL /well	overnight @ 4˚C	1x	plate covered with titer top
All	Plate Wash	ELISA Wash Buffer[a]	-	-	20-30x	
All	Secondary Antibody	alkaline phosphatase conjugated goat anti-rabbit IgG (H+L) (1:2000 in 1X PBS)	100 µL /well	2.5-4 hr @ RT	1x	plate covered with titer top
All	Plate Wash	ELISA Wash Buffer[a]	-	-	20-30x	
All	Colormetric Label	9.8% Diethanolamine, 0.5 mM $MgCl_2$ + p-Nitrophenylphosphate[b]	100 µL /well	-	1x	
All	*Absorbance was measured at 405 nm with a Molecular Devices Spectra Max Plus microplate reader (ancient samples) or a Molecular Devices THERMOmax microplate reader (extant samples)					
	a: 1X PBS (in 18.2 MΩ UltraPure H2O) with 0.1% Tween20<br>a: 10 ml buffer (9.8% Diethanolamine, 0.5 mM MgCl2) +1 tablet p-Nitrophenylphosphate					

2. Supplemental Results

2.1 X-ray Diffraction (XRD)

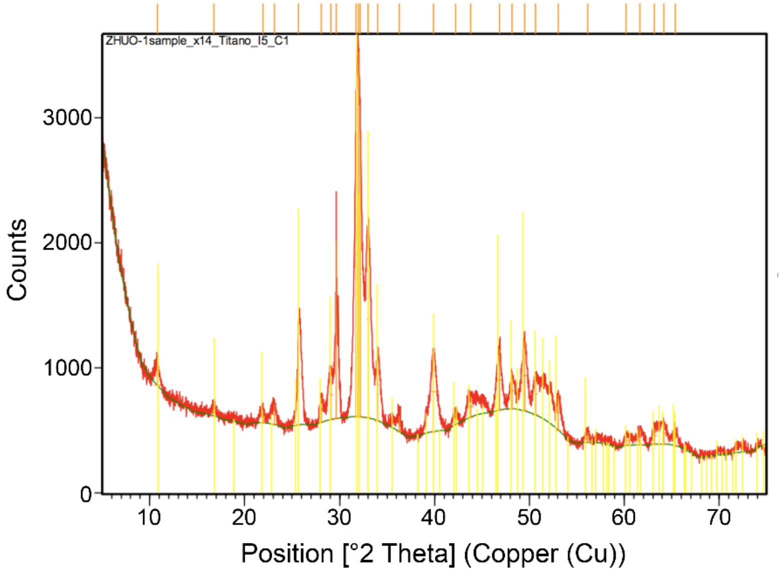

Figure S1. XRD diffractogram. X-ray diffraction (XRD) pattern obtained from analysis of sample from MPM-PV 1156-49. All peaks shown correspond with the diffraction traces of four minerals: dolomite (calcite), hydroxyapatite, fluoroapatite, and chloroapatite (Table S10).

2.2 REE Analyses

Raw transect data are provided separately in **Data S1** as an Excel XLSX file.

2.2.1 Sources for Environmental Data in Figure 7.

Literature data for environmental samples in Figure 7 of the main text are as follows: river waters (green field; Hoyle et al., 1984; Goldstein and Jacobsen, 1988; Elderfield et al., 1990; Bau and Dulski, 1996; Biddau et al., 2002; Åström and Corin, 2003; Bwire Ojiambo et al., 2003; Tang et al., 2003; Centeno et al., 2004; Johannesson et al., 2004; Gammons et al., 2005a, 2005b; Barroux et al., 2006; Bau et al., 2006; Kulaksiz and Bau, 2007, 2011b; Pokrovsky et al., 2010; Censi et al., 2015; Kalender and Aytimur, 2016; Smith and Liu, 2018); suspended river loads (dull pink field; Goldstein and Jacobsen, 1988; Merschel et al., 2017); groundwaters (bright pink field; Smedley, 1991; Johannesson et al., 1995, 1997, 1999; Leybourne et al., 2000; Bwire Ojiambo et al., 2003; Tang and Johannesson, 2006; Vardanjani et al., 2012; Chevis et al., 2015; Liu et al., 2016); lakes (purple field; Johannesson and Lyons, 1995; Johannesson et al., 1995, 2004; Leybourne et al., 2000; De Carlo and Green, 2002; Bwire Ojiambo et al., 2003; Gammons et al., 2005a; Bau et al., 2006); estuaries (yellow field; Elderfield et al., 1990; Sholkovitz, 1993; Nozaki et al., 2000; Chevis et al., 2015; Rousseau et al., 2015); coastal waters (light blue field; Hoyle et al., 1984; Elderfield and Sholkovitz, 1987; Elderfield et al., 1990; Bau and Dulski, 1996; Kulaksiz and Bau, 2007; Bayon et al., 2011); seawater (dark blue field; Elderfield and Greaves, 1982; De Baar et al., 1983; German et al., 1991; Piepgras and Jacobsen, 1992; Sholkovitz et al., 1994; German et al., 1995; Zhang and Nozaki, 1996; Hongo et al., 2006; Wang

and Yamada, 2007; van de Flierdt et al., 2012; Grenier et al., 2013; Jeandel et al., 2013; Garcia-Solsona et al., 2014; Abbott et al., 2015; Hathorne et al., 2015; Zheng et al., 2016; Johannesson et al., 2017; Osborne et al., 2017; de Baar et al., 2018); sea floor particles (gray field; Sholkovitz et al., 1994; Garcia-Solsona et al., 2014); marine pore fluids (orange field; Elderfield and Sholkovitz, 1987; Haley et al., 2004; Kim et al., 2011; Johannesson et al., 2017).

2.2.2 Supplemental REE Results Figures

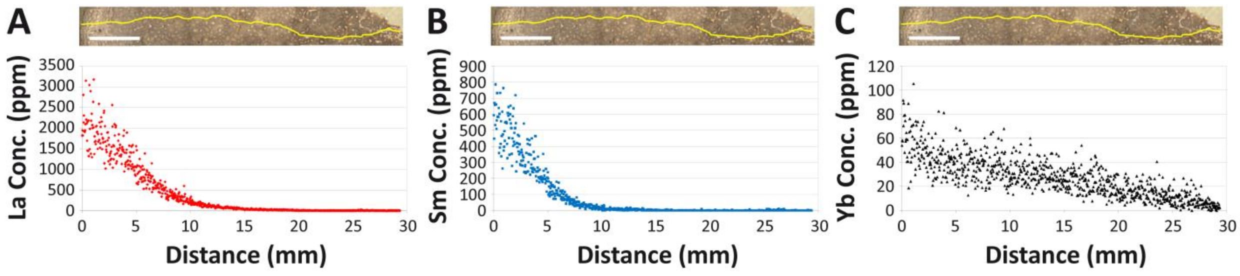

Figure S2. Intra-bone REE concentration gradients of various elements in *Dreadnoughtus.* Intra-bone REE concentration gradients of various elements in the left humerus of *Dreadnoughtus* (MPM-PV 1156-49). (**A**) The light REE (LREE) lanthanum (La). (**B**) The middle REE (MREE) samarium (Sm). (**C**) The heavy REE (HREE) ytterbium (Yb). Note that each panel has different concentration scales. Yellow line at the top of each panel depicts the track of the laser across the bone thick section during analyses. Scale bars (in white over bone images) each equal 1 mm.

2.3 Soft Tissue Preservation

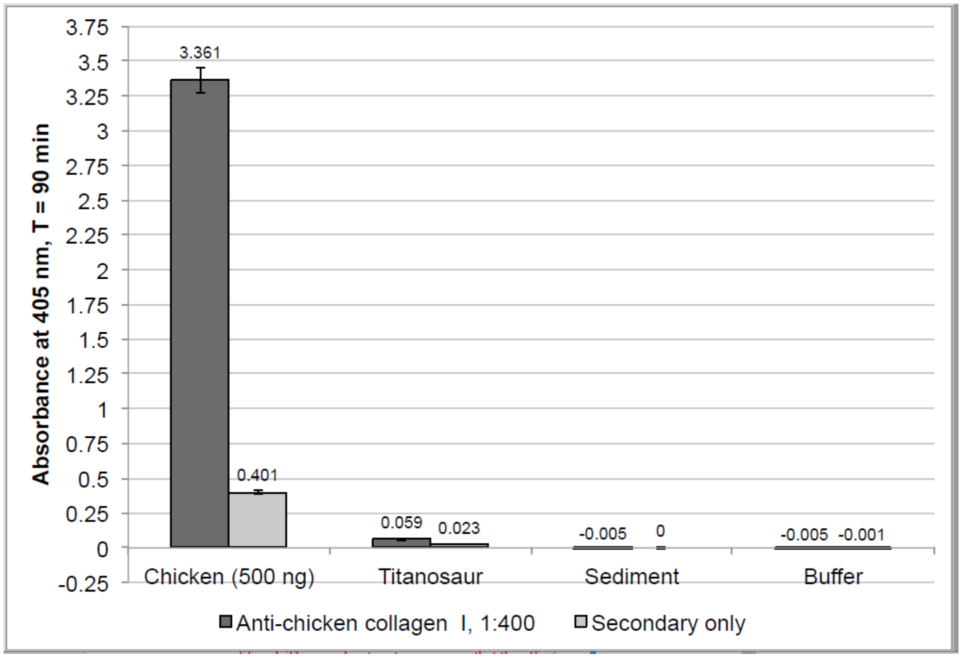

Figure S3. ELISA results of chicken bone compared to *Dreadnoughtus.* Enzyme-linked immunosorbent assay (ELISA) results of chemical extractions from extant chicken bone (500 ng of extraction products per well), Dreadnoughtus schrani bone, sediment, and buffer (extraction products from 150 mg of bone or sediment per well). Dark grey columns (left) represent absorbance (405 nm, time = 90 min) obtained for extractions incubated with anti-collagen I antibodies (1:400). Light grey columns (right) show secondary-only control for each sample. When 500 ng of chicken extraction products were plated, absorbance reached saturation (3.0+) within 90 minutes. At 90 minutes, D. schrani extracts incubated with anti-collagen I antibodies showed substantially less absorbance (~0.060) than chicken, but still exceeded the threshold value of twice the background control (~0.023). indicating a positive detection of collagen I. Sediment and buffer extractions showed no absorbance, indicating that signal observed for D. schrani is not the result of contamination from the burial environment or laboratory. Error bars represent one standard deviation above and below mean absorbance values for each sample.

3. Supplemental Discussion

3.1 Potential Tetrad Effects in MPM-PV 1156-49

Lack of MREE enrichment causes transects across the cortex of MPM-PV 1156-49 to lack distinct W-shape NASC-normalized profiles indicative of tetrad effects (unique elemental behavior based on ionic charge, radius, and configuration of the outer electron shell; Hinz and Kohn, 2010; Herwartz et al., 2013) or extensive secondary apatite precipitation (Herwartz et al., 2013). However, a couple transects across the middle cortex display potential evidence of weak tetrad effects in the form of very subtle W-shape NASC normalized patterns (Figure 5B), positive Y/Ho anomalies (Figure S2; cf. Herwartz et al., 2013), and/or increasingly positive $(La/La^*)_N$ anomalies with cortical depth (Figure S2; cf., Herwartz et al., 2013). According to Herwartz et al. (2013), these trends may reflect either: (1) tetrad effects during uptake; (2) fractionation during uptake, in which LREE are preferentially adsorbed by the external-most cortex (resulting in changing of the pore fluid composition as it percolates deeper into the bone), and/or; (3) incorporation of elemental readings from secondarily precipitated (authigenic) phosphate which has scavenged trace elements from the pore fluid (i.e., during recrystallization of bone crystallites into larger, more diagenetically-stable fluorapatite crystals; cf. Hubert et al., 1996). Although it is difficult to tease apart the relative importance of these potential causes, the complete lack of any signs of double medium diffusion (*sensu* Kohn, 2008) through Haversian canals in addition to advective diffusion in concentration-depth profiles (Table 2) implies that fractionation during uptake was likely a major contributing factor. Abundant other data (normal intra-bone fractional trends in the spider diagram [Figure 6B], the flatter profile for U than REE despite their similar diffusivities [Figure 4A,B], low concentrations of MREE and elements with low-moderate diffusivities in middle cortices [Figure 6A,B, Data S1]) also signify fractionation during uptake and changes in the pore fluid composition over time. Therefore, the positive Y/Ho and $(La/La^*)_N$ anomalies observed in the middle cortex of MPM-PV 1156-49 may reflect this typical “fossilization” process more so than tetrad effects.

3.2 Intra-Bone Fractionation in $(La/Yb)_N$ vs. $(La/Sm)_N$

The pattern seen in the femur of MOR 1125 of decreasing $(La/Yb)_N$ yet increasing $(La/Sm)_N$ with cortical depth (Figure 7 of main text) has been interpreted many ways in past reports. For example, Reynard et al. (1999) assigned such patterns to represent "substitution-recrystallization" REE incorporation mechanics, whereas Trueman et al. (2006) interpreted such specimens (their 'group 4') to reflect long-term REE scavenging by adsorption. Most recently, Herwartz et al. (2013) reconsidered $(La/Yb)_N$–$(La/Sm)_N$ patterns in light of new laser-ablation derived data and concluded that all previous interpretations based on solution ICPMS analyses (e.g., Reynard et al., 1999; Trueman et al., 2006) were biased by bulk sample averaging effects. Herwartz et al. (2013, p. 176) (and Trueman et al., 2011) offer a compelling argument that such ratio trends reflect "'normal' intra-bone fractionation" driven by decreasing partition coefficients with increasing atomic radii for REE; because the order of closest fit of REE ionic radii to the Ca^{2+} lattice site in bone hydroxyapatite is $Sm^{3+} > La^{3+} > Yb^{3+}$, fractionation based on ionic radius will occur during uptake, causing preferential earlier uptake of Sm and La by the external cortex because of their better fit. This "normal" fractionation would thus simultaneously depress $(La/Yb)_N$ values and raise $(La/Sm)_N$ values in the internal cortex relative to the external cortex, as seen in MPM-PV 1156-49 (Figure 7B). If the majority of uptake with fractionation occurred during early diagenesis, as most evidence appears to support, then the bone composition likely reflects uptake from circum-neutral pH groundwaters (cf., Martin et al., 2005).

REFERENCES

Abbott, A.N.; Haley, B.A.; McManus, J.; Reimers, C.E. The sedimentary flux of dissolved rare earth elements to the ocean. *Geochim. Cosmochim. Ac*. **2015**, *154*, 186–200.

Åström, M.; Corin, N. Distribution of rare earth elements in anionic, cationic and particulate fractions in boreal humus-rich streams affected by acid sulphate soils. *Water Res.* **2003**, *37*, 273–280.

Barroux, G.; Sonke, J.E.; Boaventura, G.; Viers, J.; Godderis, Y.; Bonnet, M.-P.; Sondag, F.; Gardoll, S.; Lagane, C.; Seyler, P. Seasonal dissolved rare earth element dynamics of the Amazon River main stem, its tributaries, and the Curuaí floodplain. *Geochem. Geophy. Geosy.* **2006**, *7*, Q12005.

Bau, M.; Dulski P. Anthropogenic origin of positive gadolinium anomalies in river waters. *Earth Planet. Sc. Lett*. **1996**, *143*, 245–255.

Bau, M.; Knappe, A.; Dulski, P. Anthropogenic gadolinium as a micropollutant in river waters in Pennsylvania and in Lake Erie, northeastern United States. *Chem. Erde-Geochem.* **2006**, *66*, 143–152.

Bayon, G.; Birot, D.; Ruffine, L.; Caprais, J.-C.; Ponzevera, E.; Bollinger, C.; Donval, J.-P.; Charlou, J.-L.; Voisset, M.; Grimaud, S. Evidence for intense REE scavenging at cold seeps from the Niger Delta margin. *Earth Planet. Sc. Lett*. **2011**, *312*, 443–452.

Biddau, R.; Cidu, R.; Frau, F. Rare earth elements in waters from the albitite-bearing granodiorites of Central Sardinia, Italy. *Chem. Geol*. **2002**, *182*, 1–14.

Bwire Ojiambo, S.; Lyons, W.B.; Welch, K.A.; Poreda, R.J.; Johannesson, K.H. Strontium isotopes and rare earth elements as tracers of groundwater-lake water interactions, Lake Naivasha, Kenya. *Appl. Geochem.* **2003**, *18*, 1789–1805.

Censi, P.; Sposito, F.; Inguaggiato, C.; Zuddas, P.; Inguaggiato, S.; Venturi, M. Zr, Hf and REE distribution in river water under different ionic strength conditions. *Sci. Total Environ.* **2018**, *645*, 837–853.

Centeno, L.M.; Faure, G.; Lee, G.; Talnagi, J. Fractionation of chemical elements including the REEs and ^{226}Ra in stream contaminated with coal-mine effluent. *Appl. Geochem.* **2004**, *19*, 1085–1095.

Chevis, D.A.; Johannesson, K.H.; Burdige, D.J.; Tang, J.; Moran, S.B.; Kelly, R.P. Submarine groundwater discharge of rare earth elements to a tidally-mixed estuary in Southern Rhode Island. *Chem. Geol*. **2015**, *397*, 128–142.

de Baar, H.J.W.; Bruland, K.W.; Schijf, J.; van Heuven, S.M.A.C.; Behrens, M.K. Low cerium among the dissolved rare earth elements in the central North Pacific Ocean. *Geochim. Cosmochim. Ac*. **2018**, *236*, 5–40.

de Baar, H.J.W.; Bacon, M.P.; Brewer, P.G. Rare-earth distributions with a positive Ce anomaly in the Western North Atlantic Ocean. *Nature* **1983**, *301*, 324–327.

De Carlo, E.H.; Green W.J. Rare earth elements in the water column of Lake Vanda, McMurdo Dry Valleys, Antarctica. *Geochim. Cosmochim. Ac*. **2002**, *66*, 1323–1333.

Elderfield, H.; Greaves, M.J. The rare earth elements in seawater. *Nature* **1982**, *296*, 214–219.

Elderfield, H.; Sholkovitz, E.R. Rare earth elements in the pore waters of reducing nearshore sediments. *Earth Planet. Sc. Lett*. **1987**, *82*, 280–288.

Elderfield, H.; Upstill-Goddard, R.; Sholkovitz, E.R. The rare earth elements in rivers, estuaries, and coastal seas and their significance to the composition of ocean waters. *Geochim. Cosmochim. Ac*. **1990**, *54*, 971–991.

Esmaeili-Vardanjani, M.; Shamsipour-Dehkordi, R.; Eslami, A.; Moosaei, F.; Pazand, K. A study of differentiation pattern and rare earth elements migration in geochemical and hydrogeochemical environments of Airekan and Cheshmeh Shotori areas (Central Iran). *Environ. Earth Sci.* **2013**, *68*, 719-732.

Gammons, C.H.; Wood, S.A.; Pedrozo, F.; Varekamp, J.C.; Nelson, B.J.; Shope, C.L.; Baffico, G. Hydrogeochemistry and rare earth element behavior in a volcanically acidified watershed in Patagonia, Argentina. *Chem. Geol*. **2005a**, *222*, 249–267.

Gammons, C.H.; Wood, S.A.; Nimick, D.A. Diel behavior of rare earth elements in a mountain stream with acidic to neutral pH. *Geochim. Cosmochim. Ac*. **2005b**, *69*, 3747–3758.

Garcia-Solsona, E.; Jeandel, C.; Labatut, M.; Lacan, F.; Vance, D.; Chavagnac, V.; Pradoux, C. Rare earth elements and Nd isotopes tracing water mass mixing and particle-seawater interactions in the SE Atlantic. *Geochim. Cosmochim. Ac*. **2014**, *125*, 351–372.

German, C.R.; Masuzawa, T.; Greaves, M.J.; Elderfield, H.; Edmond, J.M. Dissolved rare earth elements in the Southern Ocean: cerium oxidation and the influence of hydrography. *Geochim. Cosmochim. Ac*. **1995**, *59*, 1551–1558.

German, C.R.; Holliday, B.P.; Elderfield, H. Redox cycling of rare earth elements in the suboxic zone of the Black Sea. *Geochim. Cosmochim. Ac*. **1991**, *55*, 3553–3558.

Goldstein, S.J.; Jacobsen, S.B. Rare earth elements in river waters. *Earth Planet. Sc. Lett*. **1988**, *89*, 35–47.

Grenier, M.; Jeandel, C.; Lacan, F.; Vance, D.; Venchiarutti, C.; Cros, A.; Cravatte, S. From the subtropics to the central equatorial Pacific Ocean: neodymium isotopic composition and rare earth element concentration variations. *J. Geophys. Res.-Oceans* **2013**, *118*, 592–618.

Haley, B.A.; Klinkhammer, G.P.; McManus, J. Rare earth elements in pore waters of marine sediments. *Geochim. Cosmochim. Ac*. **2004**, *68*, 1265–1279.

Hathorne, E.C.; Stichel, T.; Brück, B.; Frank, M. Rare earth element distribution in the Atlantic sector of the Southern Ocean: the balance between particle scavenging and vertical supply. *Mar. Chem.* **2015**, *177*, 157–171.

Herwartz, D.; Tütken, T.; Jochum, K.P.; Sander, P.M. Rare earth element systematics of fossil bone revealed by LA-ICPMS analysis. *Geochim. Cosmochim. Ac*. **2013**, *103*, 161–183.

Hinz, E.A.; Kohn, M.J. The effect of tissue structure and soil chemistry on trace element uptake in fossils. *Geochim. Cosmochim. Ac*. **2010**, *74*, 3213–3231.

Hongo, Y.; Obata, H.; Alibo, D.S.; Nozaki, Y. Spatial variations of rare earth elements in North Pacific surface water. *J. Oceanogr.* **2006**, *62*, 441–455.

Hoyle, J.; Elderfield, H.; Gledhill, A.; Greaves, M. The behaviour of the rare earth elements during mixing of river and sea waters. *Geochim. Cosmochim. Ac*. **1984**, *48*, 143–149.

Hubert, J.F.; Panish, P.T.; Chure, D.J.; Prostak, K.S. Chemistry, microstructure, petrology, and diagenetic model of Jurassic dinosaur bones, Dinosaur National Monument, Utah. *J. Sediment. Res*. **1996**, *66*, 531–547.

Jeandel, C.; Delattre, H.; Grenier, M.; Pradoux, C.; Lacan, F. Rare earth element concentrations and Nd isotopes in the Southeast Pacific Ocean. *Geochem. Geophy. Geosy.* **2013**, *14*, 328–341.

Johannesson, K.H.; Palmore, C.D.; Fackrell, J.; Prouty, N.G.; Swarzenski, P.W.; Chevis, D.A.; Telfeyan, K.; White, C.D.; Burdige, D.J. Rare earth element behavior during groundwater-seawater mixing along the Kona Coast of Hawaii. *Geochim. Cosmochim. Ac*. **2017**, *198*, 229–258.

Johannesson, K.H.; Tang, J.; Daniels, J.M.; Bounds, W.J.; Burdige, D.J. Rare earth element concentrations and speciation in organic-rich blackwaters of the Great Dismal Swamp, Virginia, USA. *Chem. Geol*. **2004**, *209*, 271–294.

Johannesson, K.H.; Farnham, I.M.; Guo, C.; Stetzenbach, K.J. Rare earth element fractionation and concentration variations along a groundwater flow path within a shallow, basin-fill aquifer, southern Nevada, USA. *Geochim. Cosmochim. Ac*. **1999**, *63*, 2697–2708.

Johannesson, K.H.; Stetzenbach, K.J.; Hodge, V.F. Rare earth elements as geochemical tracers of regional groundwater mixing. *Geochim. Cosmochim. Ac*. **1997**, *61*, 3605–3618.

Johannesson, K.H.; Lyons, W.B.; Stetzenbach, K.J.; Byrne, R.H. The solubility control of rare earth elements in natural terrestrial waters and the significance of PO_4^{3-} and CO_3^{2-} in limiting dissolved rare earth concentrations: a review of recent information. *Aquat. Geochem.* **1995a**, *1*, 157–173.

Johannesson, K.H.; Lyons, W.B. Rare-earth element geochemistry of Colour Lake, an acidic freshwater lake on Axel Heiberg Island, Northwest Territories, Canada. *Chem. Geol*. **1995b**, *119*, 209–223.

Kalender, L.; Aytimur, G. REE geochemistry of Euphrates River, Turkey. *J. Chem.* **2016**, *1*, 1–13.

Kim, J.-H.; Torres, M.E.; Haley, B.A.; Kastner, M.; Pohlman, J.W.; Riedel, M.; Lee, Y.J. The effect of diagenesis and fluid migration on rare earth element distribution in fluids of the northern Cascadia accretionary margin. *Chem. Geol*. **2011**, *291*, 152–165.

Kohn, M.J. Models of diffusion-limited uptake of trace elements in fossils and rates of fossilization. *Geochim. Cosmochim. Ac*. **2008**, *72*, 3758–3770.

Kulaksiz, S.; Bau, M. Anthropogenic gadolinium as a microcontaminant in tap water used as drinking water in urban areas and megacities. *Appl. Geochem.* **2011**, *26*, 1877–1885.

Kulaksiz, S.; Bau, M. Contrasting behaviour of anthropogenic gadolinium and natural rare earth elements in estuaries and the gadolinium input into the North Sea. *Earth Planet. Sc. Lett*. **2007**, *260*, 361–371.

Leybourne, M.I.; Goodfellow, W.D.; Boyle, D.R.; Hall, G.M. Rapid development of negative Ce anomalies in surface waters and contrasting REE patterns in groundwaters associated with Zn-Pb massive sulphide deposits. *Appl. Geochem.* **2000**, *15*, 695–723.

Liu, H.; Guo, H.; Xing, L.; Zhan, Y.; Li, F.; Shao, J.; Niu, H.; Liang, X.; Li, C. Geochemical behaviors of rare earth elements in groundwater along a flow path in the North China Plain. *J. Asian Earth Sci.* **2016**, *117*, 33–51.

Martin, J.E.; Patrick, D.; Kihm, A.J.; Foit, F.F.; Grandstaff, D.E. Lithostratigraphy, tephrochronology, and rare earth element geochemistry of fossils at the classical Pleistocene Fossil Lake area, south central Oregon. *J. Geol*. **2005**, *113*, 139–155.

Merschel, G.; Bau, M.; Schmidt, K.; Münker, C.; Dantas, E.L. Hafnium and neodymium isotopes and REY distribution in the truly dissolved, nanoparticulate/colloidal and suspended loads of rivers in the Amazon Basin, Brazil. *Geochim. Cosmochim. Ac*. **2017**, *213*, 383–399.

Nozaki, Y.; Lerche, D.; Alibo, D.S.; Tsutsumi, M. Dissolved indium and rare earth elements in three Japanese rivers and Tokyo Bay: evidence for anthropogenic Gd and In. *Geochim. Cosmochim. Ac*. **2000**, *64*, 3975–3982.

Osborne, A.H.; Hathorne, E.C.; Schijf, J.; Plancherel, Y.; Böning, P.; Frank, M. The potential of sedimentary foraminiferal rare earth element patterns to trace water masses in the past. *Geochem. Geophy. Geosy.* **2017**, *18*, 1550–1568.

Piepgras, D.J.; Jacobsen, S.B. The behavior of rare earth elements in seawater: precise determination of variations in the North Pacific water column. *Geochim. Cosmochim. Ac*. **1992**, *56*, 1851–1862.

Pokrovsky, O.S.; Viers, J.; Shirokova, L.S.; Shevchenko, V.P.; Filipov, A.S.; Dupré, B. Dissolved, suspended, and colloidal fluxes of organic carbon, major and trace elements in the Severnaya Dvina River and its tributary. *Chem. Geol*. **2010**, *273*, 136–149.

Reynard, B.; Lecuyer, C.; Grandjean, P. Crystal-chemical controls on rare-earth element concentrations in fossil biogenic apatites and implications for paleoenvironmental reconstructions. *Chem. Geol*. **1999**, *155*, 233–241.

Rousseau, T.C.C.; Sonke, J.E.; Chmeleff, J.; van Beek, P.; Souhat, M.; Boaventura, G.; Seyler, P.; Jeandel, C. Rapid neodymium release to marine waters from lithogenic sediments in the Amazon estuary. *Nat. Commun.* **2015**, *6*, 7592.

Sholkovitz, E.R. The geochemistry of rare earth elements in the Amazon River estuary. *Geochim. Cosmochim. Ac*. **1993**, *57*, 2181–2190.

Sholkovitz, E.R.; Landing, W.M.; Lewis, B.L. Ocean particle chemistry: the fractionation of rare earth elements between suspended particles and seawater. *Geochim. Cosmochim. Ac*. **1994**, *58*, 1567–1579.

Smedley, P.L. The geochemistry of rare earth elements in groundwater from the Carnmenellis area, southwest England. *Geochim. Cosmochim. Ac*. **1991**, *55*, 2767–2779.

Smith, C.; Liu, X.-M. Spatial and temporal distribution of rare earth elements in the Neuse River, North Carolina. *Chem. Geol*. **2018**, *488*, 34–43.

Tang, J.; Johannesson, K.H. Controls on the geochemistry of rare earth elements along a groundwater flow path in the Carrizo Sand aquifer, Texas, USA. *Chem. Geol*. **2006**, *225*, 156–171.

Tang, J.; Johannesson, K.H. Speciation of rare earth elements in natural terrestrial waters: assessing the role of dissolved organic matter from the modeling approach. *Geochim. Cosmochim. Ac*. **2003**, *67*, 2321–2339.

Trueman, C.N.; Kocsis, L.; Palmer, M.R.; Dewdney, C. Fractionation of rare earth elements within bone mineral: a natural cation exchange system. *Palaeogeogr. Palaeocl*. **2011**, *310*, 124–132.

Trueman, C.N.; Behrensmeyer, A.K.; Potts, R.; Tuross, N. High-resolution records of location and stratigraphic provenance from the rare earth element composition of fossil bones. *Geochim. Cosmochim. Ac*. **2006**, *70*, 4343–4355.

Ullmann, P. V.; Grandstaff, D.E.; Ash, R.D.; Lacovara, K.J. Geochemical taphonomy of the Standing Rock Hadrosaur Site: Exploring links between rare earth elements and cellular and soft tissue preservation. *Geochim. Cosmochim. Acta***2020**, *269*, 223–237, doi:10.1016/j.gca.2019.10.030.

van de Flierdt, T.; Pahnke, K.; Amakawa, H.; Andersson, P.; Basak, C.; Coles, B.; Colin, C.; Crocket, K.; Frank, M.; Frank, N.; Goldstein, S.L.; Goswami, V.; Haley, B.A.; Hathorne, E.C.; Hemming, S.R.; Henderson, G.M.; Jeandel, C.; Jones, K.; Kreissig, K.; Lacan, F.; Lambelet, M.; Martin, E.E.; Newkirk, D.R.; Obata, H.; Pena, L.; Piotrowski, A.M.; Pradoux, C.; Scher, H.D.; Schöberg, H.; Singh, S.K.; Stichel, T.; Tazoe, H.; Vance, D.; Yang, J. GEOTRACES intercalibration of neodymium isotopes and rare earth element concentrations in seawater and suspended particles. Part 1: reproducibility of results for the international intercomparison. *Limnol. Oceanogr. Methods* **2012**, *10*, 234–251.

Werner, M.; Chott, A.; Fabiano, A.; Battifora, H. Effect of formalin tissue fixation and processing on immunohistochemistry. *Am. J. Surg. Pathol.* **2000**, *24*, 1016–1019, doi:10.1097/00000478-200007000-00014.

Wang, Z.-L.; Yamada, M. Geochemistry of dissolved rare earth elements in the Equatorial Pacific Ocean. *Environ. Geol.* **2007**, *52*, 779–787.

Zhang, J.; Nozaki, Y. Rare earth elements and yttrium in seawater: ICP-MS determinations in the East Caroline, Coral Sea, and South Fiji basins of the western South Pacific Ocean. *Geochim. Cosmochim. Ac*. **1996**, *60*, 4631–4644.

Zheng, W.; Schweitzer, M.H. Chemical Analyses of Fossil Bone. In *Forensic Microscopy for Skeletal Tissues: Methods and Protocols*; Bell, L.S., Ed.; Humana Press: Totowa, New Jersey, 2012; pp. 153–172 ISBN 978-1-61779-976-1.

Zheng, X.-Y.; Plancherel, Y.; Saito, M.A.; Scott, P.M.; Henderson, G.M. Rare earth elements (REEs) in the tropical South Atlantic and quantitative deconvolution of their non-conservative behavior. *Geochim. Cosmochim. Ac*. **2016**, *177*, 217–237.
